# *Helicobacter pylori* genetic diversification in the Mongolian gerbil model

**DOI:** 10.7717/peerj.4803

**Published:** 2018-05-18

**Authors:** Amber C. Beckett, John T. Loh, Abha Chopra, Shay Leary, Aung Soe Lin, Wyatt J. McDonnell, Beverly R.E.A. Dixon, Jennifer M. Noto, Dawn A. Israel, Richard M. Peek Jr, Simon Mallal, Holly M. Scott Algood, Timothy L. Cover

**Affiliations:** 1 Department of Pathology, Microbiology and Immunology, Vanderbilt University School of Medicine, Nashville, TN, United States of America; 2 Department of Medicine, Vanderbilt University School of Medicine, Nashville, TN, United States of America; 3 Institute for Immunology and Infectious Diseases, Murdoch University, Murdoch, Australia; 4 Tennessee Valley Healthcare System, Veterans Affairs, Nashville, TN, United States of America

**Keywords:** *Helicobacter pylori*, Quasispecies, Mutation, Genetic diversity, Evolution, Animal models

## Abstract

*Helicobacter pylori* requires genetic agility to infect new hosts and establish long-term colonization of changing gastric environments. In this study, we analyzed *H. pylori* genetic adaptation in the Mongolian gerbil model. This model is of particular interest because *H. pylori*-infected gerbils develop a high level of gastric inflammation and often develop gastric adenocarcinoma or gastric ulceration. We analyzed the whole genome sequences of *H. pylori* strains cultured from experimentally infected gerbils, in comparison to the genome sequence of the input strain. The mean annualized single nucleotide polymorphism (SNP) rate per site was 1.5e^−5^, which is similar to the rates detected previously in *H. pylori-*infected humans. Many of the mutations occurred within or upstream of genes associated with iron-related functions (*fur*, *tonB1*, *fecA2*, *fecA3*, and *frpB3*) or encoding outer membrane proteins (*alpA, oipA, fecA2, fecA3, frpB3* and *cagY*). Most of the SNPs within coding regions (86%) were non-synonymous mutations. Several deletion or insertion mutations led to disruption of open reading frames, suggesting that the corresponding gene products are not required or are deleterious during chronic *H. pylori* colonization of the gerbil stomach. Five variants (three SNPs and two deletions) were detected in isolates from multiple animals, which suggests that these mutations conferred a selective advantage. One of the mutations (FurR88H) detected in isolates from multiple animals was previously shown to confer increased resistance to oxidative stress, and we now show that this SNP also confers a survival advantage when *H. pylori* is co-cultured with neutrophils. Collectively, these analyses allow the identification of mutations that are positively selected during *H. pylori* colonization of the gerbil model.

## Introduction

*Helicobacter pylori* is a Gram-negative, microaerophilic spiral-shaped bacterium that colonizes the gastric mucosa of approximately 50% of the global human population (Atherton & Blaser, 2009; Cover & Blaser, 2009; Kusters, Van Vliet & Kuipers, 2006; Suerbaum & Michetti, 2002). While most individuals colonized with *H. pylori* never develop any adverse effects, the presence of *H. pylori* is a strong risk factor for gastric cancer, peptic ulcer disease, and iron deficiency anemia (Cover & Blaser, 2009; Ernst & Gold, 2000; Kusters, Van Vliet & Kuipers, 2006; Suerbaum & Michetti, 2002).

Among individuals colonized with *H. pylori*, the risk of gastric cancer is higher in those who are colonized with *H. pylori* strains secreting proteins that cause alterations in host cells (such as the oncoprotein CagA translocated through a type IV secretion stystem, and s1/i1/m1 forms of the VacA toxin) than in those colonized with strains that lack CagA and produce other forms of VacA  (Cover, 2016; Hatakeyama, 2014). The consumption of diets with a high salt content, low iron content, or low content of fruits and vegetables is an additional risk factor for gastric cancer (Cover & Peek, 2013). Several host genetic factors (e.g., certain polymorphisms of the interleukin-1β gene) also influence gastric cancer risk (El-Omar et al., 2000; Figueiredo et al., 2002).

*H. pylori* strains isolated from unrelated humans exhibit a high level of genetic diversity (Dorer, Sessler & Salama, 2011; Gressmann et al., 2005; Linz et al., 2014; Suerbaum & Josenhans, 2007). This diversity is attributable to a high mutation rate and a high rate of intraspecies recombination (Cao et al., 2015; Dorer, Sessler & Salama, 2011; Linz et al., 2014; Suerbaum & Josenhans, 2007). Previous studies have examined *H. pylori* genetic diversification in individual human stomachs over time or during *H. pylori* transmission to new human hosts, and have demonstrated that the mutation rate is particularly high during transmission to new hosts (Didelot et al., 2013; Israel et al., 2001; Kennemann et al., 2011; Linz et al., 2013; Linz et al., 2014).

Genetic diversification of *H. pylori* has also been detected during infection of animal models (Barrozo et al., 2013; Barrozo et al., 2016; Behrens et al., 2013; Hansen et al., 2017; Loh et al., 2015; Noto et al., 2017; Solnick et al., 2004; Yamaoka & Graham, 2014). The Mongolian gerbil model is of particular interest because *H. pylori*-infected gerbils develop severe gastric inflammation, sometimes accompanied by gastric cancer and/or gastric ulceration (Franco et al., 2005; Gaddy et al., 2013; Noto et al., 2013; Ogura et al., 2000; Watanabe et al., 1998; Wirth et al., 1998). Since Mongolian gerbils are outbred, the genetic variation in these animals mirrors the genetic variation that occurs among human hosts. Therefore, the gerbil model can potentially be used to correlate specific disease states with *H. pylori* mutation rates or accumulation of specific mutations.

Several previous studies have analyzed *H. pylori* diversification in the Mongolian gerbil model (Behrens et al., 2013; Farnbacher et al., 2010; Loh et al., 2015; Noto et al., 2017). One previous study reported that a FurR88H mutation was detected more commonly in strains isolated from gerbils fed a high salt diet than in strains from gerbils fed a regular diet (Loh et al., 2015). *In vitro* experiments indicated that the FurR88H mutation conferred resistance to high salt conditions and oxidative stress (Loh et al., 2015). It was proposed that a high-salt diet promotes high levels of gastric inflammation and oxidative stress in gerbils infected with *H. pylori*, and that these conditions, along with high levels of intraluminal sodium chloride, lead to selection of *H. pylori* strains that are most fit for growth in this environment (Loh et al., 2015). Recently, the FurR88H mutation was also detected more commonly in *H. pylori* strains cultured from gerbils maintained on a low-iron diet than in strains cultured from gerbils maintained on an iron-replete diet, and was detected more commonly among isolates from humans with premalignant lesions than in isolates from humans with non-atrophic gastritis alone (Noto et al., 2017).

In the current study, we sought to identify additional mutations that are positively selected during *H. pylori* colonization of the Mongolian gerbil model. To do this, we analyzed the genome sequences of *H. pylori* strains isolated from experimentally infected gerbils that were fed different diets and that exhibited diverse outcomes of infection (Beckett et al., 2016). The variations in diet and disease outcome in these animals mirror the variations in diet and disease outcome that are observed in humans colonized with *H. pylori*. We then used stringent criteria to identify genetic variations that were present in the strains cultured from gerbils compared to the input strain, and we calculated a mean mutation rate over the time course of the study. Most of the SNPs identified in output strains corresponded to non-synonymous mutations, and several of these were detected in output strains from multiple animals. We show that one of the SNPs detected in output strains from multiple animals, FurR88H, confers a survival advantage when *H. pylori* is co-cultured with neutrophils. We also identified deletion or insertion mutations that disrupted open reading frames in the output strains, suggesting that the corresponding gene products are either not required or deleterious during chronic *H. pylori* colonization of the gerbil stomach.

## Materials and Methods

### *H. pylori* colonization of Mongolian gerbils

*H. pylori* strain B128 was isolated from a human with gastric ulceration (McClain et al., 2009). *H. pylori* strain 7.13 is an *in vivo*-adapted strain that was isolated from a Mongolian gerbil infected with *H. pylori* strain B128. Unlike the parental strain B128, the output *H. pylori* strain 7.13 reproducibly includes cancer in the Mongolian gerbil model (Franco et al., 2005). The *H. pylori* strains analyzed in this study were isolated from a previously described cohort of Mongolian gerbils that were infected with strain 7.13, using a protocol approved by the Vanderbilt University IACUC (protocol M/14/021) (Beckett et al., 2016). Briefly, male gerbils (aged 3–5 weeks) were fed one of three diets: a normal diet (Test Diet AIN-93M, Purina Mills), a high salt diet (modified to contain 8.25% salt compared to 0.25% salt in the normal AIN-93M diet) or a low iron diet (manufactured to contain 0 ppm iron compared to 39 ppm iron in the normal AIN-93M diet) (Beckett et al., 2016). After receiving these diets for a period of two weeks, gerbils received two orogastric inoculations with *H. pylori* strain 7.13 (Beckett et al., 2016). At 16 weeks post-infection, gastric tissue was collected and *H. pylori* was cultured as described previously (Beckett et al., 2016). The analyses of gastric pH, gastric histology and hematologic parameters in this cohort of gerbils have been described previously (Beckett et al., 2016). A pool of colonies isolated from each gerbil was frozen at −70 °C until the time when genome sequence analysis was undertaken.

### Isolation of *H. pylori* chromosomal DNA

*H. pylori* output strains cultured from gerbils were minimally passaged on trypticase soy agar plates containing 5% sheep blood (Hemostat Laboratories, Dixon, CA, USA), and then were streaked for single colony isolation. Individual colonies were isolated and expanded by growth on separate plates. Bacteria harvested from one-day-old plates were resuspended in 1 ml of phosphate buffered saline, and genomic DNA was isolated using a Wizard Genomic purification kit (Promega, Madison, WI, USA) and eluting the DNA into water.

### *H. pylori* genome sequencing

*H. pylori* DNA samples were subjected individually to enzymatic fragmentation using the NEBNext™ dsDNA Fragmentase kit (NEB) according to the manufacturer’s instructions, with average fragment length of 600 bp (range of 400–1,000 bp). Libraries of DNA were prepared from purified fragmented DNA samples using the Kapa Hyper Prep library kit with unique indexes as per the manufacturer’s protocol (Kapa Biosystems, Inc. Wilmington, MA, USA). Quantification of these library preps was performed with the Kapa library quantification kit (Kapa Biosystems, Inc. Wilmington, MA, USA), and sequenced on a MiSeq sequencer using the 600V3 kit (Illumina Inc., San Diego, USA). For the analysis, raw reads were quality trimmed and aligned to the reference sequence (*H. pylori* strain B8) (Farnbacher et al., 2010) using CLCbio Genomics workbench version 8.5 (Table S1). Data pertaining to read counts, fold coverage, and percent unmapped reads are shown in the Table S1. Alignment parameters were as follows: Mismatch cost = 2, Insertion cost = 3, Deletion cost = 3, Insertion open cost = 6, Insertion extend cost = 1, Deletion open cost = 6, Deletion extend cost = 1, Length fraction = 0.5, Similarity fraction = 0.8. The alignment files exported from the CLCbio workbench in BAM format were then imported into an in-house-developed application (VGAS) for further coverage and SNP analysis. SNP reports were generated using a 10% cut-off, and genome-wide comparisons of SNPs in the output strains compared to the input strain were then performed. Sequence data were deposited in NCBI (Bioproject ID: PRJNA414609).

Three single *H. pylori* colonies cultured from each gerbil were isolated, expanded and sequenced individually, and three colonies of the input strain were also sequenced in the same manner. All *H. pylori* sequence data from each animal (three single colonies per animal) were analyzed as a group, and all of the sequence reads of the input strain (3 single colonies) were analyzed as a group. We sought to identify polymorphisms that were detected in ≥75% of sequence reads of output strains from individual animals, and ≤10% of sequence reads from the input strain. This approach allowed identification of polymorphisms that were maximally different when comparing output strain populations with the input strain. Mean annualized SNP rates per site were determined by calculating a ratio of the total number of SNPs in each strain to the genome size (based on the colonization of gerbils for 16 weeks), and then multiplying the values by 3.25 to approximate the number of mutations anticipated to arise over a period of one year.

### McDonald–Kreitman analysis

We performed the McDonald–Kreitman test (McDonald & Kreitman, 1991) on nucleotide sequences of the genes of interest in output strains from each gerbil, relative to the input strain. Synonymous changes were used as the neutral class to test the hypothesis that these genes maintained their sequences in a neutral fashion via mutation and random genetic drift. Consensus sequences for each output strain were derived excluding polymorphisms representing <10% of the bases at a given position, and alignments of these consensus sequences to the reference genome were performed using the MUSCLE algorithm with default parameters. As the McDonald–Kreitman test is generalizable to noncoding DNA elements (Andolfatto, 2008), we also assessed the codon neutrality of the noncoding regions upstream of two genes (*fecA2* and *katA*), where SNPs were detected at high frequency. We also performed a separate multi-locus McDonald–Kreitman test to assess the evenness of positive selection across these regions using the Mantel-Haenszel test, as previously described (Egea, Casillas & Barbadilla, 2008). Finally, as the McDonald–Kreitman test is subject to Type I error, we used Bonferroni correction to adjust the *p*-values of the individual tests.

### Preparation of murine neutrophils and co-culture with *H. pylori*

Murine neutrophils were isolated using a protocol approved by the Vanderbilt University IACUC (protocol V/15/130). Briefly, using a 21-gauge needle, 1 mL of sterile casein solution was injected into the peritoneal cavity of each mouse. An inflammatory response was allowed to develop overnight, and a second dose of casein solution was administered the following morning. Animals were euthanized 3 h after the second injection. The abdominal skin was sterilized with 70% ethanol and retracted to expose the intact peritoneal wall. The peritoneal cavity was then filled with 5 mL of sterile PBS, using a 25-gauge needle, and the abdomen massaged. The fluid was slowly removed using a 25-gauge needle, placed in a 50 mL conical flask and the procedure repeated a second time. The pooled peritoneal fluid was centrifuged for 10 min at 200× g, followed by red blood cell lysis using Ammonium-Chloride-Potassium lysing buffer (ACK; Gibco, Waltham, MA, USA). Peritoneal exudates were washed 3 times, resuspended in 1 mL media (F-12 with 5% FBS) and cell numbers were counted. Finally, the cell solution was brought to the desired concentration (5  × 10^5^ cells/mL) and 1 mL was distributed to each well of 12-well cell culture plates. Plates were incubated for 1 h prior to addition of *H. pylori*.

*H. pylori* strain 7.13 (encoding wild-type Fur) and an isogenic mutant (encoding FurR88H) were tagged with distinct antibiotic resistance markers (chloramphenicol or kanamycin resistance), as described previously (Loh et al., 2015). Overnight cultures of these strains were inoculated into separate fresh broth cultures (Brucella broth containing 5% FBS) and allowed to grow for 6 h. Murine neutrophils were then co-cultured with the *H. pylori* strains at an estimated multiplicity of infection (MOI) of 20:1 (based on measurement of OD_600_), either individually or in competition experiments. In parallel, mock infections were carried out by addition of *H. pylori* to tissue culture medium (F-12 medium containing fetal bovine serum) alone. In the competition experiments, a 1:1 mixture of *H. pylori* strains producing WT Fur or FurR88H were co-cultured with the neutrophils. Following a 1 h co-culture, the samples were treated with saponin (0.1% final concentration) (Kwok et al., 2002). Dilutions of the saponin-treated samples were plated on Brucella agar plates containing the appropriate antibiotics, and CFUs were counted 5 days after plating.  The survival of strains co-cultured with neutrophils was compared to the survival of the same strains in medium alone to calculate percent survival. Wilcoxon matched-pairs signed rank test was used to compare the survival of WT strains with survival of FurR88H mutant strains.

### Analysis of catalase enzymatic activity

Overnight broth cultures of *H. pylori* were inoculated into fresh broth cultures and grown to an OD_600_ of ∼0.5–0.6. Samples were normalized to an OD_600_ of 0.1, and catalase enzymatic assays were then performed with the Amplex red catalase kit (Life Technologies). To measure catalase activity, the culture samples were serially diluted 2-fold, and the catalase activity of the diluted cultures was compared to that of purified catalase standards provided in the Amplex red catalase kit (Life Technologies, Carlsbad, CA, USA). Enzymatic measurements were performed in accordance with the manufacturer’s instructions.

## Results

### Identification of single nucleotide polymorphisms (SNPs)

To gain a better understanding of how *H. pylori* adapts to different gastric environments, we investigated the genetic diversification of *H. pylori* that occurs during colonization of Mongolian gerbils. We analyzed *H. pylori* strains isolated 16 weeks post-infection from a previously described cohort of gerbils (Beckett et al., 2016). To maximize the number of *H. pylori* genetic adaptions detected, we analyzed *H. pylori* strains cultured from five gerbils that were fed different diets and that exhibited substantial variation in gastric pathology (Beckett et al., 2016). One animal (Gerbil #1) was maintained on a normal diet and had severe disease (defined as high inflammation scores, gastric cancer and ulcer, increased gastric pH and/or anemia) (Table 1). Two gerbils (Gerbil #2 and #3) were maintained on a high salt diet; Gerbil #2 had severe disease and Gerbil #3 had less severe disease (defined as relatively low inflammation scores, lack of gastric cancer and ulcers, normal pH and not anemic) (Table 1). Gerbils #4 and #5 were maintained on a low iron diet; Gerbil #5 had severe disease and Gerbil #4 had less severe disease (Table 1). Three individual *H. pylori* colonies isolated from each gerbil (total of 15 single colony isolates, designated as output strains), as well as three individual colonies of the input strain, were analyzed by whole genome sequencing. We then identified differences in the genomes of the output strains compared to the genome of the input strain, using the stringent criteria described in the Methods, which were designed to identify genetic changes that occurred in response to strong selective pressure.

**Table 1:** Characteristics of individual gerbils.^a^

Gerbil	Diet	Hemoglobin^b^	Gastric pH	Gastric ulcer	Gastric inflammation score^c^	Gastric cancer	Mutation rate^d^
1	Normal	10.7	4.0	Yes	12	Yes	1.16E−05
2	High salt	10.6	4.5	Yes	12	Yes	1.55E−05
3	High salt	12.5	3.0	No	6.5	No	7.77E−06
4	Low iron	14.0	3.0	No	6.5	No	9.71E−06
5	Low iron	10.9	7.0	Yes	11	Yes	3.11E−05

**Notes.**

a Gerbils were fed the indicated diets and euthanized 16 weeks after *H. pylori* infection.

b Hemoglobin values indicate g/dl.

c Gastric inflammation was scored on a scale from 0 to 12.

d Mean annualized SNP rate per site.

**Figure 1:**
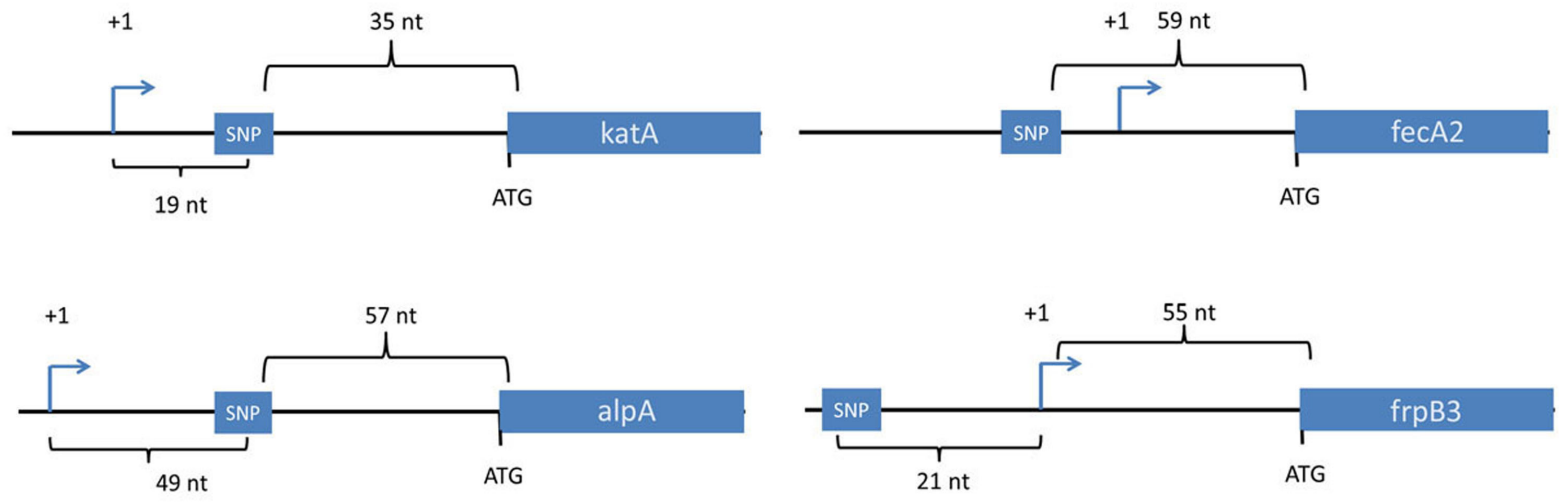
Location of SNPs in non-coding regions. Four SNPs in non-coding regions were mapped in the context of nearby genes. The transcriptional start sites for these genes were mapped previously based on use of differential RNA-seq methodology or primer extension analysis  (Danielli et al., 2009; Sharma et al., 2010). All four SNPs were within 60 nucleotides of a downstream gene, and two were downstream of transcriptional start sites. Transcriptional start sites are labeled as +1. Nt, the number of nucleotides between the depicted genetic elements. Two of the SNPs (upstream of *fecA2* and *katA*) were present in all of the output strains, but not the input strain.

Collectively, the output strains contained 25 unique SNPs that were either not detected or detected at very low levels in the input strain (Table 2). Twenty-one were in coding regions and four were in non-coding regions (Table 2). All 4 of the SNPs in non-coding regions (Table 2) were localized less than 60 nucleotides upstream of a translational start site (*katA*, *fecA2*, *frpB3*, and *alpA*) (Table 2 and Fig. 1). Two of these were downstream of transcriptional start sites, within 5′ untranslated regions of the mRNA (*katA* and *alpA*) (Fig. 1). Among the 21 mutations in coding regions, 17 were non-synonymous and 4 were synonymous (Table 2). Four of the non-synonymous SNPs were in *cagY*, which encodes a component of the type IV secretion system that translocates the CagA effector protein into host cells. Two of the non-synonymous changes in *cagY* were instances in which a sense codon was mutated to a stop codon. The SNPs in *cagY* were all identified in output strains from the same animal (Gerbil #5), and were localized within regions that are repeated multiple times within the gene. Mapping the precise sites of such mutations within repeat regions is challenging using the sequencing technology used in this study. We did not conduct additional studies to verify the precise sites of the *cagY* mutations listed in Table 2.

**Table 2:** Single nucleotide polymorphisms detected in *H. pylori* strains cultured from gerbils.

Location^a^	Gerbil identification numbers^b^	SNP description	Percent of output reads with SNP^c^	Percent of input reads with SNP	Nucleotide change (5′->3′)^d^	MKT positive selection *p* value^e^
Non-coding region (Base Position 23384)	1	Upstream of *frpB3*	100	<2	G->T	0.29
Hypothetical Protein (HPB8_343, Base Position 313249)	1	Non-Synonymous Thr to Ile (AA#60)	100	<2	C->T	0.23
Non-coding region (Base Position 613051)	1	Upstream of *alpA*	100	<5	G->A	0.31
*cysS* (Base Position 641561)	1	Non-Synonymous Val to Ile (AA#12)	97	<2	G->A	0.71
Non-coding region (Base Position 1001780)	1, 2, 3, 4, 5	Upstream of *fecA2*	99,98,99,99,99	<3	C->T	0.01
Non-coding region (Base Position 1064871)	1, 2, 3, 4, 5	Upstream of *katA*	96,96,96,97,97	<6	C->G	0.01
Hypothetical Protein (HPB8_45, Base Position 52225)	2	Non-Synonymous Ser to Gly (AA#160)	100	<1	A->G	0.64
Hypothetical Protein (HPB8_64, Base Position 71813)	2	Non-Synonymous Gly to STOP (AA#51)	100	<4	C->T	0.50
Hypothetical Protein (HPB8_593, Base Position 563527)	2	Non-Synonymous Glu to Lys (AA#54)	100	<1	G->A	0.44
*fur* (Base Position 1122559)	2, 3, 4, 5	Non-Synonymous Arg to His (AA#88)	98,99,99,99	<2	G->A	0.03
*rpoD* (Base Position 1449954)	2	Synonymous(AA#533)	100	<3	G->A	0.72
Hypothetical Protein (HPB8_32, Base Position 38960)	5	Non-Synonymous Tyr to His (AA#145)	100	<1	T->C	0.24
*nadD* (Base Position, 135265)	5	Non-Synonymous Pro to Leu (AA#152)	100	<2	C->T	0.92
*folE* (Base Position 587541)	4	Non-Synonymous Phe to Leu (AA#52)	100	<2	T->C	0.90
*cagN* (Base Position 679850)	5	Non-Synonymous Pro to His (AA#125)	100	<2	C->A	0.33
*cagY* (Base Position 693086)	5	Synonymous (AA#1006)	88	<3	A->G	0.97
*cagY* (Base Position 693101)	5	Synonymous (AA#1011)	100	<10	G->A	0.58
*cagY* (Base Position 693132)	5	Non-Synonymous Gln to Glu (AA#1022)	100	<8	C->G	0.41
*cagY* (Base Position 693219)	5	Non-Synonymous Lys to STOP (AA#1051)	100	<5	A->T	0.37
*cagY* (Base Position 693226)	5	Non-Synonymous Leu to STOP (AA#1053)	100	<9	T->A	0.18
*cagY* (Base Position 693240)	5	Non-Synonymous Val to Leu (AA#1058)	86	<6	G->C	0.67
*thrB* (Base Position 1145232)	5	Synonymous (AA#192)	100	<3	G->A	0.42
*hcpE* (Base Position 1302755)	5	Non-Synonymous Gly to Ser (AA#241)	100	<2	G->A	0.68
*glmM* (Base Position 1462161)	5	Non-Synonymous Ala to Thr (AA#149)	100	<2	G->A	0.13
*cheV7* (Base Position 1572769)	5	Non-Synonymous Ala to Val (AA#298)	100	<3	C->T	0.16

**Notes.**

a Base positions in the genome of reference strain B8 are listed.

b *H. pylori* output strains cultured from the indicated animals contained the designated SNPs, based on criteria defined in Methods. See Table 1 for description of animals.

c The mean percent of reads containing the designated SNP, based on sequence analysis of three individual *H. pylori* colonies cultured from each animal. Multiple values are listed if the SNP was detected in *H. pylori* isolates from multiple animals.

d The nucleotide changes listed are relative to the ORF of the indicated genes.

e Positive selection was analyzed using the McDonald Kreitman test.

The identification of predominantly non-synonymous SNPs in the output strains supports the hypothesis that these mutations were positively selected. Formal testing of this hypothesis is difficult due to the small number of strains analyzed, but we nevertheless performed a McDonald Kreitman analysis to compare the genome sequences of three single colony isolates of the input strain compared to the output strains. This analysis provided evidence that the FurR88H SNP and two mutations in non-coding regions were positively selected (Table 2).

In a previous study, we analyzed the genome sequences of *H. pylori* strains cultured from two gerbils using 454 sequencing methods (Loh et al., 2015). Five SNPs were detected in 100% of sequence reads of isolates from the animal on a high salt diet, but were not detected (or detected in low abundance) in the input strain or isolates from the animal on a regular diet (Loh et al., 2015). Two of these SNPs were identified in multiple output strains in the current study. Specifically, a mutation upstream of *fecA2* was identified in all 5 output strains in the current study, and a FurR88H mutation was identified in 4 of the 5 output strains (Table 2). The only output strain that did not have the FurR88H mutation was isolated from a gerbil consuming a normal diet.

**Figure 2:**
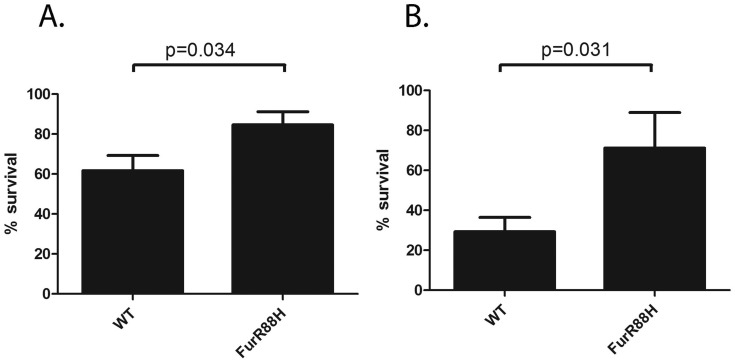
FurR88H confers a survival advantage to *H. pylori* when co-cultured with neutrophils. To determine the effect of the FurR88H mutation on bacterial survival in the presence of neutrophils, we co-cultured strain 7.13 producing wild-type (WT) Fur and an isogenic mutant producing FurR88H, each labeled with a different antibiotic resistance marker, with freshly isolated murine neutrophils. (A) Neutrophils were co-cultured individually with either the strain producing WT Fur or an isogenic mutant producing FurR88H. A total of 10 independent biological replicates of each *H. pylori*-neutrophil co-culture sample (from 6 sets of experiments) were used in this analysis. In the competition experiment shown in (B), a 1:1 mixture of *H. pylori* strains producing WT Fur or FurR88H were cocultured with the neutrophils. A total of six independent biological replicates of such co-cultures (from two experiments) were used for this analysis. The survival of *H. pylori* strains co-cultured with neutrophils was quantified by analysis of CFU/ml, as described in the Methods, and was compared to the survival of the same strains in media alone, to determine % survival. When cultured individually with neutrophils, the strain producing FurR88H had a significantly higher percent survival compared to strains producing wild-type Fur (*p* = 0.034, student’s *t*-test) (A). In competition assays (B), strains producing FurR88H showed a higher survival compared to strains producing wild-type Fur (*p* = 0.031, Wilcoxon matched-pairs signed rank test).

Fur is a regulatory protein that controls gene expression in response to iron availability (Pich & Merrell, 2013). We showed previously that the FurR88H mutation confers increased resistance to high concentrations of salt or conditions of oxidative stress (Loh et al., 2015). Resistance to oxidative stress would presumably provide an important selective advantage in the context of the *H. pylori*-induced gastric mucosal inflammatory response, which is characterized by an infiltration of neutrophils, macrophages, and other immune cell types. To further define how the FurR88H mutation might confer a selective advantage in the gastric environment, we conducted studies in which isogenic *H. pylori* strains producing wild-type Fur or FurR88H (each strain harboring a different antibiotic marker) were co-cultured with neutrophils. We detected a significant survival advantage of the isogenic mutant strain containing the FurR88H mutation, compared to wild-type strain (Fig. 2). These data indicate that the FurR88H mutation confers a survival advantage to *H. pylori* in a neutrophil-containing environment.

**Figure 3:**
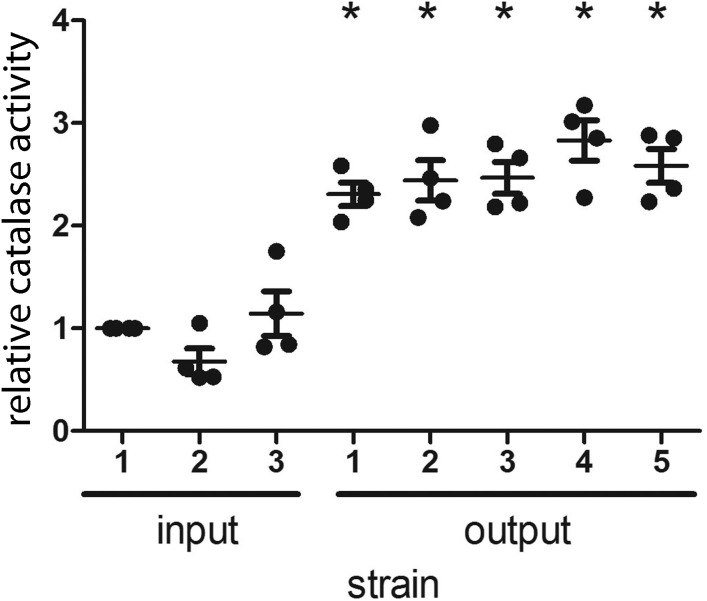
*H. pylori* strains cultured from gerbils demonstrate increased catalase enzymatic activity compared to the input strain. Three sequenced single colony isolates of the input strain and a representative sequenced single colony isolate of each output strain were tested for catalase activity, as described in Methods. Each data point represents the mean catalase activity of the strain tested, compared to input single colony isolate 1. The mean catalase activity of each strain was calculated based on four independent experiments. Gerbil output strains demonstrated increased catalase enzymatic activity compared to the input strain (*, *p* < 0.05, Mann–Whitney *U* test).

*H. pylori* catalase (encoded by *katA*) confers resistance to oxidative stress (Benoit & Maier, 2016), and catalase is essential for *H. pylori* infection of mice (Harris et al., 2003). In a previous study, we noted that output strains cultured from gerbils had markedly higher catalase activity than the input strain (Loh et al., 2015). The input strain used in the previous study contained a frameshift mutation within *katA* and many of the output strains contained an intact *katA* ORF, which accounted for the difference in catalase activity (Loh et al., 2015). In contrast to the previous study, the input strain used for the current study had an intact *katA* ORF. We analyzed the catalase activity of the output strains in the current study compared to the input strain, and found that all of the output strains had increased catalase activity compared to the input strain (Fig. 3). Further experiments will be necessary to evaluate whether this change is a consequence of a mutation downstream of the *katA* transcriptional start site (within 5′ untranslated region).

### Mutation rate

In an effort to quantify the rate of genetic change during the four months in which gerbils were colonized with *H. pylori*, we calculated the annualized SNP rate per site, as described in the Methods. Overall, the mean annualized SNP rate per site (the number of SNPs that would be expected to occur per site, over the course of one year) among all output strains was 1.5e^−5^, with a range of 7.77e^−6^ to 3.11e^−5^ among individual output strains (Table 1). Interestingly, the mutation rate detected in output strains was positively correlated with the gastric pH in the corresponding gerbils (i.e., higher numbers of SNPs were detected in strains from animals with a high gastric pH) (*r* = 0.93, Pearson correlation coefficient, *p* = 0.0204).

### Deletions and insertions

**Figure 4:**
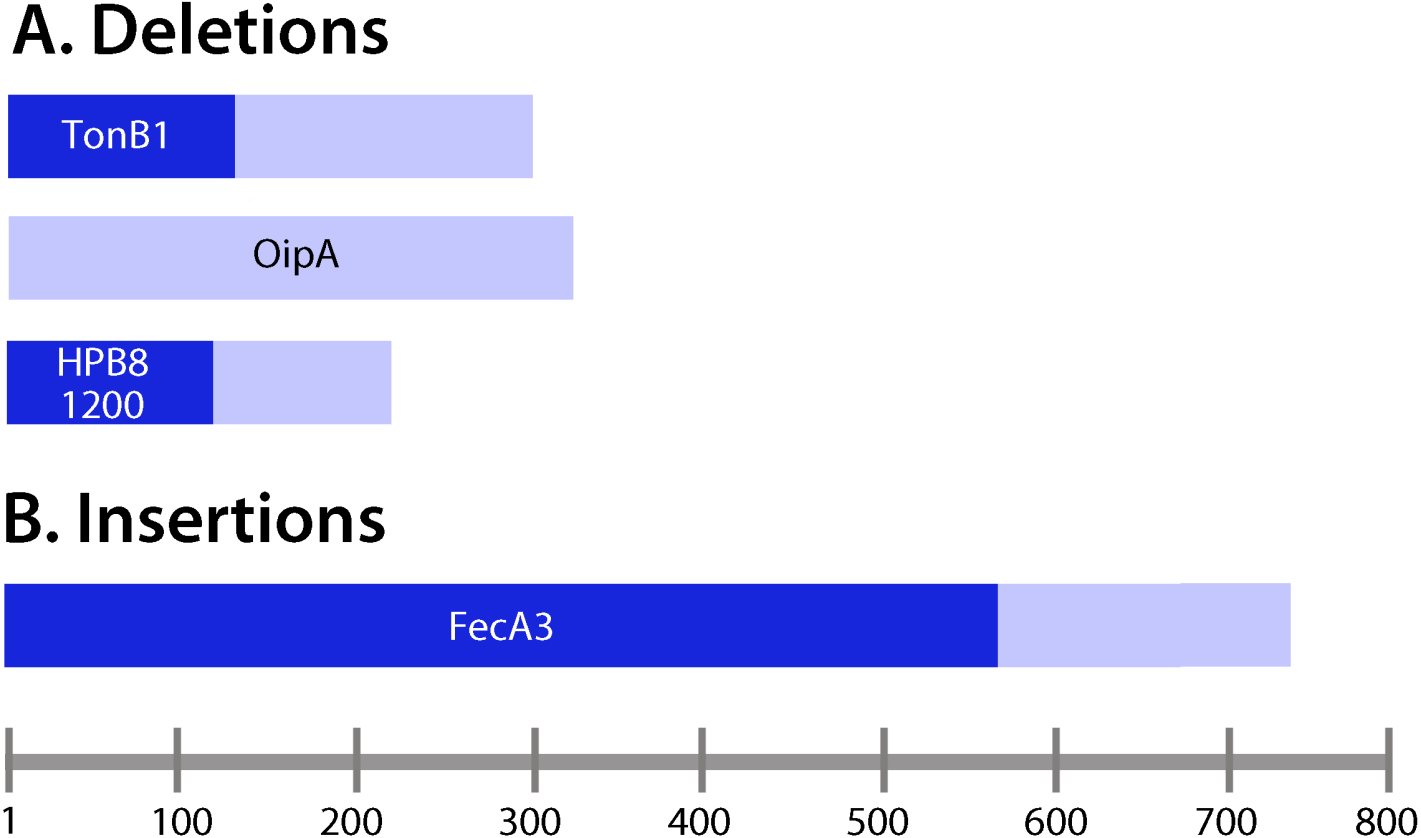
Analysis of insertions and deletions in coding regions. (A) Deletions; (B) insertions. Lengths of the deduced protein products encoded by the corresponding genes containing insertions and deletions were examined. Frameshift mutations (Table 3) were located upstream of the resulting premature stop codons in the ORFs of interest. Pale bars indicate the lengths of the protein products encoded by wild-type (non-mutated) genes, and the darker bars indicate the lengths of the proteins encoded by genes harboring insertions or deletions. For example, *tonB1* encodes a protein 291 amino acids in length in the input strain, whereas in the presence of a frameshift mutation, a protein 111 amino acids in length is encoded.

We detected five unique deletions (ranging from one to four consecutive nucleotides deleted in individual genes) and three unique insertions among the output strains (Table 3). Three of the deletions were in coding regions and two were in intergenic regions. The genes containing deletions were *oipA* (also known as *hopH*, encoding an outer membrane protein), *tonB1* (encoding a protein required for activity of outer membrane receptors involved in iron acquisition), and a gene encoding a hypothetical protein (Table 3). The intergenic region deletions were upstream of genes encoding an LPS 1,2-glucosyltransferase and a hypothetical protein. Among the deletions in coding regions, all were frameshift mutations. One of the insertions was in a coding region and two were in intergenic regions. The insertion in a coding region was in the gene encoding the outer membrane protein FecA3*,* and it was a frameshift mutation. Fifty percent of the deletions or insertions occurred within polynucleotide tracts (Table 3). All of the insertions or deletions (indels) that occurred in coding regions resulted in protein truncation (Fig. 4).

**Table 3:** Insertions and deletions detected in *H. pylori* strains cultured from gerbils.

Location^a^	Gerbil identification numbers^b^	Percent of input reads with indel	Percent of output reads with indel^c^	Indel type	Polynucleotide tract?^d^
*tonB1* (132930–132931)	1, 4, 5	0	76,76,76	Deletion	No
Upstream of hypothetical protein (Base Position 217342)	1	0	80	Deletion	T(14)
*oipA* (819621–819624)	1	0	90	Deletion	GA(9)
Hypothetical protein (HPB8_1200, Base Position 1171489)	1, 5	0	100,100	Deletion	T(9)
Intergenic region between a membrane protein and LPS 1,2-glucosyltransferase (Base Position 1332441)	2	0	80	Deletion	T(17)
Upstream of membrane protein (WP_013195952.1, Base Position 1584449)	1	0	75	Insertion (C)	No
*fecA3* (Base Position 1661940)	2	0	96	Insertion (T)	No
Upstream of chemotaxis protein HPB8_1462 (Base Position 1432303)	4	0	80	Insertion (G)	No

**Notes.**

a Base positions in the genome of reference strain B8 are listed.

b *H. pylori* output strains cultured from the indicated numbers of animals contained the designated indels.

c The mean percent of reads containing the designated indel, based on sequence analysis of 3 individual *H. pylori* colonies cultured from each animal. Multiple values are listed if the indel was detected in *H. pylori* isolates from multiple animals.

d The tables shows characteristics of polynucleotide tracts in the input strain.

### Detection of genetic changes in multiple animals

Most of the SNPs were detected in *H. pylori* isolates from only one of the gerbils analyzed, but 3 were detected in isolates from multiple animals (four or five gerbils) (Tables 2 and 4). The FurR88H mutation discussed earlier was detected in output strains from four of the five animals. Two SNPs (in non-coding regions upstream of *fecA2* and upstream of *katA*) were found in output strains isolated from all five animals (Tables 2 and 4, Fig. 1).

Most of the indels were detected in *H. pylori* isolates from a single animal, but two deletions were detected in isolates from multiple animals. Specifically, a two-nucleotide in-frame deletion in *tonB1* was detected in isolates from three animals (Tables 3 and 4). Additionally, a one base pair deletion in a gene encoding a hypothetical protein (HPB8_1200) was detected in two animals (Tables 3 and 4). The presence of SNPs or indels in strains cultured from multiple animals suggests that these mutations conferred a selective advantage.

**Table 4:** Mutations detected in *H. pylori* strains cultured from multiple gerbils^a^.

**SNPs in strains from multiple gerbils**	**Number of output strains containing mutation**^b^	**Gerbil identification numbers**
Non-coding region (Base Position 1001780)	5/5	1, 2, 3, 4, 5
Non-coding region (Base Position 1064871)	5/5	1, 2, 3, 4, 5
*Fur* (Base Position 1122559)	4/5	2, 3, 4, 5
**Deletions in strains from multiple gerbils**	**Number of output strains containing mutation**	
*tonB1* (Base Position 132930-132931)	3/5	1, 4, 5
Hypothetical protein (HPB8_1200, Base Position 1171489)	2/5	1, 5

**Notes.**

a Base positions in the genome of reference strain B8 are listed.

b *H. pylori* output strains cultured from the indicated numbers of animals contained the designated SNPs or deletions.

## Discussion

In this study, we examined *H. pylori* genetic diversification in the gastric environment of Mongolian gerbils. To maximize the number of genetic alterations detected, we analyzed *H. pylori* strains cultured from multiple different gastric environments, including the stomachs of animals fed different diets (normal, high salt or low iron), animals with different gastric pathologies (including gastric ulcer, gastric cancer, and varying severity of gastric inflammation), and animals with different hematologic parameters (either anemia or normal hemoglobin). We used stringent criteria to identify mutations that were detected in a high proportion of sequence reads from output strains and a very low proportion of sequence reads from the input strain.

The mutations detected in the output strains could have arisen *de novo* during colonization of gerbils, or alternatively, these mutations could have been present in the input strain population but not readily detectable by the sequencing approach used in this study. Recent work suggests that there can be substantial genetic diversity within individual *H. pylori* strains, consistent with the existence of a quasispecies (Draper et al., 2017; Kuipers et al., 2000). Given the high frequency with which several mutations were detected in output strains, it seems likely that many of the mutations were present in a small subpopulation of organisms in the input strain.

Many of the mutations detected in this study were likely to have been positively selected *in vivo*. The detection of predominantly non-synonymous SNPs in output strains supports this viewpoint. Use of the McDonald Kreitman test provided further evidence that several of the mutations were positively selected. Notable limitations included the small number of strains analyzed, and features of the experimental design that are not optimally compatible with assumptions on which the McDonald Kreitman test is based.

In total, we detected 25 unique SNPs, five deletions, and three insertions in output strains from at least one animal. A disproportionately high number of mutations were detected within genes or upstream of genes associated with iron-related functions (*fur*, *tonB1*, *fecA2, fecA3* and *frpB3*) or genes which encode outer membrane proteins (*alpA, oipA, fecA2, fecA3, frpB3* and *cagY*) (Pich & Merrell, 2013; Schauer et al., 2007; Voss et al., 2014; Wandersman & Delepelaire, 2004). Moreover, the genes *tonB1* and *cagY* contained multiple mutations. The large number of mutations in genes associated with iron-related functions is potentially related to the administration of modified diets (low iron or high salt) to several of the animals. Alternatively, the availability of iron in the *H. pylori*-infected gerbil stomach might be different from that in the *H. pylori*-infected human stomach.

FecA2, FecA3, and FrpB3 are outer membrane proteins predicted to be involved in iron acquisition (Voss et al., 2014). TonB1 is a transmembrane protein predicted to be involved in iron homeostasis as well as nickel import (Schauer et al., 2007; Wandersman & Delepelaire, 2004). Fur is a regulator of gene expression, particularly those genes involved in iron homeostasis, central metabolism and energy production (Pich & Merrell, 2013).

AlpA and OipA are outer membrane proteins reported to modulate *H. pylori* interactions with host cells (Dossumbekova et al., 2006; Matsuo, Kido & Yamaoka, 2017; Odenbreit et al., 1999), and CagY is a component of the *cag* type IV secretion system localized to the outer membrane (Barrozo et al., 2016; Frick-Cheng et al., 2016). The detection of mutations in genes encoding these proteins or upstream of these genes suggests that it may be beneficial for *H. pylori* to remodel its surface during colonization of the gerbil stomach, perhaps as a result of immune responses directed against specific outer membrane proteins, or as a consequence of different receptors being available in the gerbil stomach compared to the human stomach (Königer et al., 2016).

All of the insertion or deletion mutations within open reading frames were frameshift mutations predicted to result in production of truncated proteins or unstable proteins (i.e., generation of pseudogenes). One such mutation occurred in *oipA*. Analyses of *H. pylori* strains cultured from humans have shown that strains possessing a functional, in-frame *oipA* gene are associated with more severe disease outcome, such as gastric cancer or gastric ulceration, compared to strains with an out-of-frame *oipA* gene (Dossumbekova et al., 2006; Franco et al., 2008; Yamaoka et al., 2002). In the current study, the one output strain containing an *oipA* frameshift mutation was isolated from a gerbil on a normal diet that exhibited relatively severe gastric disease.

Many of the mutations detected in this study were in intergenic regions (8 of the 33 mutations). The SNPs within intergenic regions were mapped to sites upstream of *katA*, *alpA*, *fecA2* and *frpB3*. These mutations could potentially influence transcription or translation rates, or could be in small RNAs that have regulatory functions. In future studies, it will be important to examine the functional significance of these mutations.

In total, five mutations (three SNPs, two deletions) were detected in output strains cultured from multiple animals, but not the input strain. The FurR88H mutation and a mutation in *tonB1* were each detected in output strains from at least three animals. The other three mutations detected in output strains from multiple animals were SNPs in intergenic regions. The detection of these mutations in multiple animals suggests that they conferred an important selective advantage.

One of the mutations identified in output strains from multiple animals in the current study was FurR88H. This mutation was detected in output strains isolated from gerbils consuming either a low iron or high salt diet, but not the animal consuming a normal diet. We previously detected the FurR88H mutation in output strains from two different cohort of gerbils experimentally infected with *H. pylori*, and observed that it was detected more commonly in output strains from animals fed a high salt diet than in output strains from animals fed a regular diet (Loh et al., 2015), and more commonly detected in output strains from animals fed a low iron diet than in output strains from animals fed a regular diet (Noto et al., 2017). *In vitro* experiments showed that the FurR88H mutation conferred a survival advantage when *H. pylori* was cultured under conditions of oxidative stress (Loh et al., 2015). Here, we co-cultured wild-type and FurR88H strains with neutrophils, and noted that strains harboring the FurR88H mutation had a significant survival advantage. This result provides further evidence that the FurR88H mutation may enhance the ability of *H. pylori* to evade immune defenses, and may help to explain why strains harboring this mutation are able to out-compete other strains *in vivo*.

Previous studies have analyzed the microevolution of *H. pylori* in individual humans over time, during transmission to new human hosts (Didelot et al., 2013; Kennemann et al., 2011; Linz et al., 2013; Linz et al., 2014), and in animal models of infection (Barrozo et al., 2013; Behrens et al., 2013; Farnbacher et al., 2010; Linz et al., 2014; Loh et al., 2015; Noto et al., 2017). These studies have detected mutations in many genes, particularly those encoding outer membrane proteins (including BabA and other members of the Hop family) (Barrozo et al., 2013; Barrozo et al., 2016; Hansen et al., 2017; Kennemann et al., 2011; Nell et al., 2014; Solnick et al., 2004; Zhang et al., 2014). Mutations in *cagY*, which encodes a component of the *cag* T4SS, have also been detected frequently (Barrozo et al., 2013; Barrozo et al., 2016). Mutations resulting in loss of CagY production have been detected at a particularly high frequency in the mouse model of *H. pylori* infection (Barrozo et al., 2013). It is likely that mutations in genes encoding outer membrane proteins enhance the ability of the bacteria to colonize new hosts, evade the immune response, and establish persistent infection. In the current study, we detected many genetic changes in output strains similar to those reported in previous studies, including mutations in genes encoding outer membrane proteins such as OipA and CagY. Conversely, many of the mutations we detected within or upstream of genes with functions related to iron (such as *tonB1*, *fecA2*, *fecA3,* and *frpB3*) have not been commonly reported to undergo genetic adaptations during the course of chronic infection in humans.

Several previous studies have analyzed *H. pylori* genetic diversification in the gerbil model (Behrens et al., 2013; Farnbacher et al., 2010; Loh et al., 2015; Noto et al., 2017). Comparison among the studies is complicated by variations in the criteria used for identification of SNPs, but it is notable that several of the mutations detected in the current study were also detected in previous studies. For example, the FurR88H mutation was detected in output strains in two previous studies (Loh et al., 2015; Noto et al., 2017), and a mutation upstream of *fecA2* was also detected in a previous study (Loh et al., 2015).

We calculated a mean annualized SNP rate per site (the number of SNPs that would be expected to occur per site, over the course of one year) of 1.5e^−5^, with a range of 7.77e^−6^ to 3.11e^−5^ among individual output strains. One prior study examining the *H. pylori* mutation rate in chronically-infected humans detected an annualized mutation rate per site of 2.5e^−5^ (Kennemann et al., 2011), and another reported an annualized mutation rate per site of 6.1e^−4^ (Linz et al., 2014). The variation in mutation rates among studies could be due to differences in the criteria for identification of SNPs, strain differences, differences in the selective forces of individual gastric environments, or differences in phase of infection (chronic versus acute). For example, there is evidence that *H. pylori* strains colonizing humans or rhesus macaques undergo a “mutational burst” in the acute phase of infection, and then exhibit a slower mutation rate during chronic infection (Linz et al., 2014). In general, the current results suggest that *H. pylori* mutation rates in the Mongolian gerbil model are similar to the corresponding mutation rates in humans, and higher than the mutation rates observed in previous studies of *S. aureus* or *P. aeruginosa* infections in humans (Linz et al., 2014; Mwangi et al., 2007; Smith et al., 2006). Interestingly, we detected a correlation between *H. pylori* mutation rate and gastric pH (i.e., higher mutation rate in animals with elevated gastric pH). Further studies with larger numbers of animals will be required to test the reproducibility of this observation. Similarly, it will be important to test whether this relationship is also observed in *H. pylori*-infected humans.

Studies of *H. pylori* genetic diversification in animal models allow several topics to be analyzed more easily than is possible in human subjects. For example, the gerbil model is ideal for correlating *H. pylori* mutation rates or accumulation of specific mutations with specific disease states or environmental factors such as diet. A notable limitation of the current study is that we analyzed output strains from a relatively small number of animals. Therefore, the current study did not allow us to determine the effects of diet or disease state on the development of mutations. We anticipate that sequence analysis of strains from a larger number of animals will more clearly reveal correlations between specific mutations, diet and disease state. Such studies could potentially lead to the identification of biomarkers for strains associated with severe disease states. It will be important in future studies to analyze the functional consequences of the mutations that are selected during *H. pylori* colonization of the gerbil stomach under varying conditions, and to analyze the selective advantages associated with these mutations in larger populations of animals.

*H. pylori* has colonized human hosts for at least 50,000 years and is extremely well adapted to the human gastric niche (Atherton & Blaser, 2009; Moodley et al., 2012). Experimental introduction of *H. pylori* into animal models, as done in the current study, is invariably associated with disruption of this longstanding bacteria-host relationship. When *H. pylori* enters the stomach of a non-human host, there is strong selective pressure favoring the emergence of mutants that are most fit for growth in the gastric environment of the new host. We presume that some of the mutations detected in the current study reflect the adaptation of *H. pylori* to the gerbil stomach (in contrast to its natural human stomach environment). Presumably the genes that accumulated mutations abrogating protein production were not required or deleterious during chronic *H. pylori* colonization of the gerbil stomach.

*Helicobacter acinonychis*, isolated from the stomachs of large cats, is one of the species mostly closely related to *H. pylori* (Eppinger et al., 2006). It has been proposed that a common ancestor of *H. pylori* and *H. acinonychis* underwent a host jump (from humans to large cats) within the last 200,000 years, leading to the emergence of two separate species (Eppinger et al., 2006). Interestingly, many *H. pylori* genes encoding outer membrane proteins correspond to pseudogenes in *H. acinonychis*, suggesting that they were unnecessary or deleterious in stomachs of large cats, or at least not beneficial (Eppinger et al., 2006). The divergence of *H. pylori* and *H. acinonychis* over a very long time period as a consequence of a host jump correlates well with the relatively short-term results of the current study, in which several pseudogenes arose after introducing the natural human colonizer *H. pylori* into the stomach of the Mongolian gerbil.

In summary, these results provide new insights into the genetic diversification of *H. pylori* under a wide range of gastric environmental conditions. They also reveal a physiologically relevant phenotype of the commonly detected output strain mutation FurR88H in conferring a survival advantage to *H. pylori* when co-cultured with neutrophils. In addition, these studies shed new light on the genetic changes that occur when *H. pylori* is introduced into a new host species.

##  Supplemental Information

10.7717/peerj.4803/supp-1 Table S1 Supplemental Table

## Additional Information and Declarations

### Data Availability

The following information was supplied regarding data availability: All raw data is available at NCBI Bioproject Accession: PRJNA414609.
