# Supplementary material for: Helicobacter pylori genetic diversification in the Mongolian gerbil model
Source: PeerJ. 2018 May 18;6:e4803. doi: 10.7717/peerj.4803 (PMC5961626; doi:10.7717/peerj.4803)
Supplement: Table S1 [file peerj-06-4803-s001.docx]

| **Supplemental Table. Mapping Statistics** | | | | | | | | | | | |
| --- | --- | --- | --- | --- | --- | --- | --- | --- | --- | --- | --- |
| **Strain** | **Total reads** | **Mapped reads** | **Percent mapped** | **Unmapped** | **Percent unmapped** | **Average coverage** | **Standard deviation** | **Minimum excl. zero coverage regions** | **Average excl. zero coverage regions** | **Standard deviation excl. zero coverage regions** | **Percent**  **GC** |
| **Input** | 1962650 | 1786411 | 90.3 | 190423 | 9.8 | 318.4 | 35.6 | 34 | 318.4 | 35.5 | 38.8 |
| **Input** | 1835872 | 1680910 | 90.9 | 167471 | 9.2 | 299.4 | 36.0 | 2 | 299.5 | 35.9 | 38.8 |
| **Input** | 688964 | 658234 | 95.4 | 31906.5 | 4.7 | 117.8 | 15.8 | 11 | 117.8 | 15.8 | 38.8 |
| **Normal Diet High Disease** | 1171259 | 1070967 | 90.8 | 108460 | 9.3 | 190.8 | 28.5 | 1 | 191.3 | 26.9 | 38.8 |
| **Normal Diet High Disease** | 1205015 | 1113081 | 91.8 | 99921 | 8.3 | 198.4 | 28.9 | 1 | 198.9 | 27.0 | 38.8 |
| **Normal Diet High Disease** | 805806 | 759822 | 94.1 | 47587 | 6.0 | 136.0 | 21.7 | 1 | 136.4 | 20.6 | 38.8 |
| **High Salt Low Disease** | 1928457 | 1765020 | 90.9 | 176071 | 9.2 | 314.4 | 45.1 | 59 | 314.4 | 45.1 | 38.8 |
| **High Salt Low Disease** | 1976455 | 1809680 | 91.0 | 179345 | 9.1 | 322.4 | 42.9 | 30 | 322.4 | 42.9 | 38.8 |
| **High Salt Low Disease** | 664662 | 636477 | 95.6 | 29289.5 | 4.5 | 113.9 | 17.6 | 19 | 113.9 | 17.6 | 38.8 |
| **Low Iron High Disease** | 1364972 | 1222272 | 89 | 150675 | 11.1 | 217.8 | 35.0 | 1 | 217.8 | 35.0 | 38.8 |
| **Low Iron High Disease** | 453785 | 431187 | 94.9 | 23401.5 | 5.2 | 77.2 | 13.0 | 1 | 77.2 | 13.0 | 38.8 |
| **Low Iron High Disease** | 648009 | 617781 | 95.2 | 31335.5 | 4.9 | 110.6 | 16.9 | 12 | 110.6 | 16.9 | 38.8 |
| **High Salt High Disease** | 1938328 | 1788955 | 91.7 | 161841 | 8.4 | 318.8 | 39.0 | 30 | 318.8 | 39.0 | 38.8 |
| **High Salt High Disease** | 564722 | 539465 | 95.4 | 26174 | 4.7 | 96.6 | 13.9 | 7 | 96.6 | 13.9 | 38.8 |
| **High Salt High Disease** | 529203 | 504360 | 95.2 | 25750.5 | 4.9 | 90.3 | 14.0 | 9 | 90.3 | 14.0 | 38.8 |
| **Low Iron Low Disease** | 2847103 | 2449508 | 85.4 | 418139 | 14.7 | 436.2 | 64.4 | 44 | 436.2 | 64.4 | 38.8 |
| **Low Iron Low Disease** | 545241 | 523857 | 95.9 | 22355.5 | 4.1 | 93.8 | 14.8 | 13 | 93.8 | 14.8 | 38.8 |
| **Low Iron Low Disease** | 636929 | 609009 | 95.5 | 28827 | 4.6 | 109.0 | 16.6 | 13 | 109.0 | 16.6 | 38.8 |
